# Learning through instructions vs. learning through practice: flanker congruency effects from instructed and applied S-R mappings

**DOI:** 10.1007/s00426-014-0621-1

**Published:** 2014-10-22

**Authors:** Dorit Wenke, Jan De Houwer, Jeffrey De Winne, Baptist Liefooghe

**Affiliations:** 1 Department of Psychology, Humboldt University Berlin, Rudower Chaussee 18, 12489 Berlin, Germany; 2 Ghent University, Ghent, Belgium

## Abstract

We compared flanker congruency effects (FCE) for flanker stimuli that were part of merely instructed S-R mappings or S-R mappings that had already been practiced. Four new S-R mappings were instructed before each block of trials. In applied flanker blocks, each instructed stimulus could appear as target and as flanker. In merely instructed flanker blocks, two stimuli only served as targets, whereas the other two exclusively appeared as flankers. Significant FCEs were observed for both flanker conditions even though the instruction-based FCE was (a) smaller than the FCE from applied mappings and (b) decreased with task practice. These results suggest that instructions alone can induce S-R associations that lead to automatic response activation when instructed stimuli appear as flankers. Execution of instructed rules seems to strengthen the instructed associations, leading to increased response conflict.

## Introduction

The ability to quickly and flexibly link any kind of behavior to new and arbitrary environmental stimuli is one of the hallmarks of flexible human behavior (e.g., Toni & Passingham, 1999). In the laboratory, this ability is typically investigated with tasks that involve completely arbitrary stimulus-response (S-R) or category-response mappings. Unlike nonverbal animals, humans with mature and intact frontal cortices do not require extensive trial-and-error learning to acquire such mappings. Instead, they can use verbal instructions to rapidly perform as required. In particular, recent research has shown that verbal S-R instructions alone can suffice to form representations of the instructed task that allow automatic behavior from the very first trial, without prior task practice (De Houwer, Beckers, Vandorpe, & Custers, 2005; Cohen-Kdoshay & Meiran, 2007, 2009; Wenke, Gaschler, & Nattkemper, 2007; Wenke, Gaschler, Nattkemper, & Frensch, 2009; Liefooghe, Wenke, & De Houwer, 2012; Liefooghe, De Houwer, & Wenke, 2013). For example, participants in a study by Cohen-Kdoshay and Meiran (2009) were instructed to respond to different classes of stimuli (e.g., letters from the first vs. second half of the alphabet) by pressing the left or the right key. They devised an Eriksen flanker task (Eriksen & Eriksen, 1974) in which target stimuli presented at the center of the screen were flanked by either response-congruent or -incongruent distractors. In their version of the task, only a subset of the instructed stimuli actually appeared as targets in an Eriksen flanker task (Eriksen & Eriksen, 1974), and hence required enactment of the instructed mappings. The remaining stimuli exclusively served as distracting flankers. Nevertheless Cohen-Kdoshay and Meiran (2009) report a flanker congruency effect (FCE) with flankers that never served as targets. This result suggests that flankers, or the categories the flankers belonged to (e.g., first half of alphabet) automatically activated the responses assigned to them by instructions (e.g., Gratton et al., 1992), leading to fast and correct responses in the congruent condition where flankers are assigned to the same response as the target. In contrast, flankers activate a different response than the target in the incongruent condition, and thus lead to slower and more error-prone reactions to targets. Further evidence for automatic activation of merely instructed S-R mappings has been provided by studies in which instructed S-R mappings were shown to interfere with performing an independent but overlapping task (e.g., De Houwer et al., 2005; Wenke et al., 2007, 2009; Liefooghe et al., 2012, 2013).

For explanation, we (Wenke et al., 2007, 2009; also see Liefooghe et al., 2012, 2013) proposed that instructed task rules may be “translated” into a more action-based representational format (Koriat, Ben-Zur, & Nussbaum, 1990, also see Hartstra, Waszak, & Brass, 2012, for recent neuroanatomical evidence in favor of this view). Specifically, we suggested that this transformation of verbal rules may involve activating and temporary binding (e.g., Hommel, 2004) of existing conceptual codes that represent relevant features of to-be-expected stimuli and to-be-performed responses, thus establishing functional task-sets.

Instruction-based automatic effects support the “prepared reflex” metaphor (Exner, 1879; Hommel, 2000; also see Meiran, Cole, & Braver, 2012). This metaphor holds that stimuli can reflexively trigger a specified action without (much) prior practice, provided a corresponding task-set has been intentionally formed in working memory in advance to actually performing the task. The prepared-reflex metaphor in general and automatic instruction effects in particular challenge and blur the classic distinction between intentional (or controlled, algorithmic,…) S-R translation of new and arbitrary mappings, on the one hand, and automatic S-R activation (or retrieval of S-R episodes) of mappings that are either highly over-learned or involve allegedly preexisting links due to dimensional overlap, on the other hand (e.g., Logan, 1988; Kornblum, Hasbrouq, & Guiard, 1990; Anderson, 1992). In particular, they suggest that merely instructed and highly over-learned mappings may be functionally similar in that both support automatic behavior.

Nevertheless, the effects of instructions appear to be more constrained than those of actual practice. First, the effects of merely instructed mappings have been shown to depend on capacity-limited working memory. For instance, the instruction-based FCE in the study by Cohen-Kdoshay and Meiran (2007; also see Cohen, Jaudas, & Gollwitzer, 2008, for converging results obtained with a different paradigm and a different criterion for automaticity) was eliminated when working memory load was increased by adding a concurrent task to their instructed flanker task (also see Meiran & Cohen-Kdoshay, 2012). Furthermore, instruction-based automatic effects strongly depend on the intention to actually prepare and perform the instructed task. They are typically not observed when participants “merely” plan to memorize the instructed rules for later recognition or recall (Liefooghe et al., 2012), or when advance preparation does not pay off, for example, because the instructed task does not have to be executed on a large proportion of trials (Wenke et al., 2009; Liefooghe et al., 2013). By contrast, several studies conducted with already practiced mappings in dual-task settings (e.g., Hommel & Eglau, 2002), or requiring task switching (e.g., Kiesel, Wendt, & Peters, 2007; Kessler & Meiran, 2010), report evidence for automatic response activation that appears to be relatively independent of working memory load (e.g., the number of S-R mappings involved). Moreover, for practiced mappings there is evidence that previously relevant mappings continue to exert some automatic influence on ongoing behavior when no longer relevant (e.g., Yamaguchi & Proctor, 2011; Marble & Proctor, 2000; see Meiran et al., 2012, for details). It has therefore been proposed that, for instructed mappings to automatically influence behavior, task-sets need to be implemented and maintained in the capacity-limited direct access area of (procedural) working memory (Oberauer, 2010; cf. Liefooghe et al., 2012; Meiran et al., 2012). In contrast, automatic influences from already practiced mappings have been attributed to activating already existing S-R links in active long-term memory (i.e., capacity unlimited activated long-term memory according to Oberauer, 2010; cf. Meiran et al., 2012). Such a view seems consistent with learning accounts holding that repeated execution of instructed S-R mappings leads to strengthening of practiced S-R associations (e.g., Hommel, 2009), or to the formation of (qualitatively different) direct sensorimotor links (e.g., Ramamoorthy & Verguts, 2012) that do not (or no longer) require as much active maintenance of the instructed mappings in the direct access region of working memory.^1^

Despite the obvious relevance of establishing and disentangling the functional properties of automatic S-R activation by merely instructed vs. already practiced mappings, evidence regarding potential differences between the two types of mapping is so far mostly indirect. We are aware of only two studies that directly compared interference based on merely instructed and already practiced mappings on the same task to explore functional (dis)similarities between instruction-based automatic effects and automatic activation of practiced S-R mappings (Waszak, Wenke, & Brass, 2008; for a replication and supporting neuroimaging results see Brass, Wenke, Spengler, & Waszak, 2009). However, both studies failed to reveal evidence for automatic S-R activation of merely instructed mappings. Given the evidence for automatic S-R activation of merely instructed mappings found in many other studies (see above), one could argue that the latter studies did not create the right conditions for these effects to occur. For example, the high number of instructed mappings might have overtaxed the limited direct access region of working memory (see Liefooghe et al., 2012, for a comprehensive discussion). In any case, because of the lack of automatic effects of instructed S-R mappings in the Waszak et al. (2008) experiment, nothing could be concluded about the functional (dis)similarities between automatic effects of merely instructed S-R mappings and S-R mappings that have been practiced.

In sum, previous studies established that merely instructed mappings can influence behavior automatically, just like practiced mappings. Other research suggests that automatic S-R activation might functionally differ in some regards for the two types of mapping. However, evidence for functional dissimilarities between automatic effects resulting from the two types of mappings is mostly indirect so far.

### The present study

The aim of the present study was to establish and disentangle the functional properties of automatic S-R activation by merely instructed vs. already practiced mappings. This was done by directly comparing the two types of mapping in a task that has been repeatedly used to demonstrate instruction-based automatic effects. More specifically, we adapted the Eriksen flanker task that was successfully used by Cohen-Kdoshay and Meiran (2007, 2009). In our version of the task, instructions always assigned two individual stimuli to left-hand key-press responses, and another two stimuli to right-hand key-press responses. New S-R mappings were introduced on each block of trials (see Table 1 for an overview of all stimulus-sets used in the experiment). In half of the blocks, all stimuli serving as distracting flankers could also appear as targets, thus requiring a response on part of the trials. We refer to these blocks as applied flanker blocks or as the applied flanker condition. In these blocks, flankers can induce a FCE on the basis of instructions, on the basis of practice, or both. On the remaining blocks, the instructed stimuli could either appear as targets or as flankers. In this condition—referred to as the merely instructed flanker condition or as merely instructed flanker blocks—the responses assigned to the stimuli serving as flankers were never executed. Hence, an FCE in these blocks can be due only to the effects of instructions about the S-R mappings for the flankers, and not to the effect of practicing these mappings.

**Table 1:**
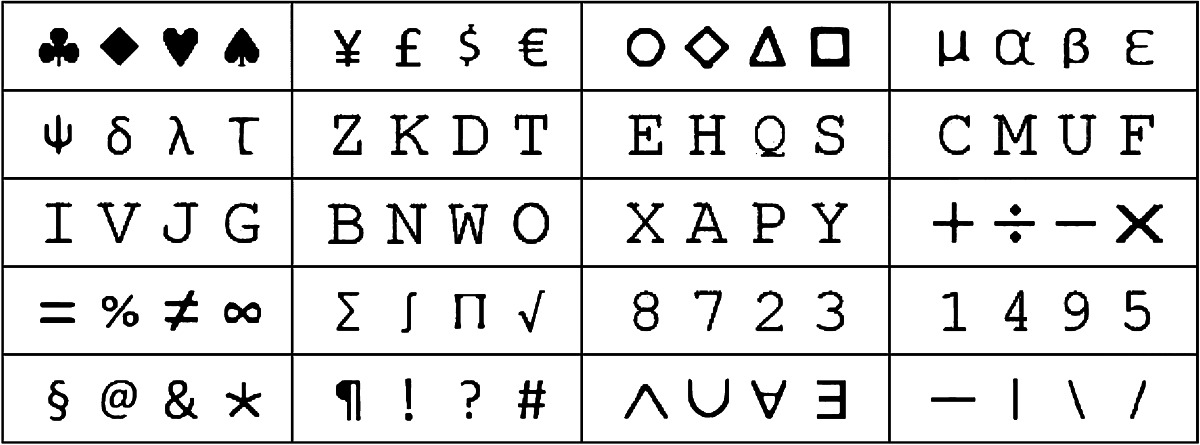
Overview of stimulus-sets

A given stimulus-set was only used in one block of trials (either in an instructed or an applied flanker block). Instructions assigned two stimuli each to left vs. right key-press responses. See text for details

With this task, we investigated (a) how flanker practice (flanker condition) affects automatic S-R activation, and hence the FCE, at comparable levels of task practice, and (b) whether the FCEs in the two flanker conditions differentially develop with task practice. We define task practice as the amount of experience with a given task (i.e., the number of trials following a given set of S-R instructions, irrespective of the nature of the targets or flankers), whereas flanker condition or flanker practice refers to the amount of experience with executing the S-R mapping for stimuli that also serve as flankers (i.e., whether or not flanker stimuli served as target stimuli on other trials, and hence were applied in the course of task practice).

#### Flanker practice

First, we wanted to ensure that evidence for automatic activation of merely instructed S-R links can be obtained with this task. To this end, we tested whether an instruction-induced FCE indicating automatic activation of instructed S-R mappings can be observed with this task, or whether flanker practice is necessary for an FCE to occur. Given the findings by Cohen-Kdoshay and Meiran (2007, 2009, 2012), we had good reasons to expect an instruction-based automatic effect in our study, thus overcoming the limitations of the studies by Waszak et al. (2008) and Brass et al. (2009). Second, we investigated whether the overall size differs between the two flanker conditions. Whereas the instruction-based FCE gives us an indication of the automatic impact of instructions on behavior, the difference between the overall FCEs in applied and merely instructed flanker blocks informs us about the effects of actually performing the instructed S-R mappings. Assuming that flanker practice leads to associative strengthening of instructed S-R mappings for flanker stimuli, we expected overall more response conflict and hence a more pronounced overall FCE in the applied flanker condition than in the merely instructed flanker condition.

#### Task practice

In addition, we investigated whether task practice differentially influences the instruction-based FCE and the execution-based FCE. The associative strengthening account predicts differential effects of task practice for merely instructed and applied flankers. In particular, we expected the instruction-based FCE to decrease with increasing task practice. Such task practice effects could arise for several reasons. First, associations based on instructed S-R mappings might dissipate over time if they are never put to use. This could happen because, over time, they become less well represented in, or excluded from, the task-set held in working memory. Second, the impact of flankers that belong to merely instructed S-R mappings could depend on the strength of the S-R associations for the target stimuli. As task practice increases, so does the number of times that the targets are responded to, and thus the strength of the S-R association for the targets (but not the flankers). In contrast, we predicted that the FCE would remain constant within applied flanker blocks in which all instructed mappings are executed equally often for a limited number of times, and hence should become similarly strengthened with task practice.

#### Sequential modulation of the FCE

Finally, using the Eriksen flanker task additionally allowed us to explore potential functional differences regarding sequential trial-by-trial modulations of the instruction-based and the execution-based FCE—the so-called Gratton effect (Gratton et al., 1992). The Gratton effect refers to the robustly observed finding with applied mappings that the FCE is larger following congruent trials (i.e., trials in which targets and flankers signal the same response) than following incongruent trials (i.e., trials in which target and flankers are assigned to different responses). One explanation of the Gratton effect, the conflict adaptation account, holds that the Gratton effect is due to flexible adjustments of cognitive control (e.g., Botvinick, Braver, Barch, Carter, & Cohen, 2001). Accordingly, the amount of response conflict encountered on a given trial determines how much control is exerted on the next trial: the more conflict, the more cognitive control (cf. Davelaar & Stevens, 2009; Wendt, Kiesel, Geringswald, Purrman, & Fischer, 2013). As outlined above, we expected more conflict from applied than from merely instructed flankers. Therefore, if conflict adaptation contributed to the sequential modulation of the FCEs in our task, one would expect a more pronounced Gratton effect in the applied than the instructed flanker condition (but see, for example, Mayr, Awh, & Laurey, 2003, for an alternative account of the Gratton effect that does not depend on response conflict; see “Discussion” for details).

## Methods

### Participants

Twenty-five students (20 women) at Ghent University took part in this study (mean age = 20.68 years) and received 8 euro for participation. All participants were native Dutch speakers and had normal or corrected to normal vision.

### Apparatus and material

The experiment was run on a computer with an Intel Core 2 Duo processor E8600 and a 19″ CRT monitor with the refresh rate set at 100 Hz. Participants were seated approximately 60 cm from the monitor. Participants responded by pressing a left or a right key (A and P keys on an AZERTY keyboard). The experiment was run using Inquisit 3.0 software (Millisecond Software). The stimuli consisted of 20 sets of 4 coherent symbols (see Table 1). Flanker-target-flanker triplets were presented horizontally and subtended 0.4° of visual angle horizontally and 0.6° of visual angle vertically. Flankers were presented within 1° of visual angle from the target. Symbol triplets were presented in black against white background in a frame at the center of the screen that subtended 11.29° of visual angle horizontally and 5.60° vertically.

### Design and procedure

The experiment consisted of 20 blocks containing 36 trials each. For each block, one of the 20 stimulus-sets was drawn without replacement (see Table 1). Two stimuli each were randomly assigned to left and right key responses.

Each block started with written instructions that stated the S-R assignments in Dutch (e.g., E and S: left key, H and Q: right key). On half of the blocks, the left key-press mappings appeared above the mappings for the right key-press response, whereas the order was reversed on the other half of the blocks. Instructions at the beginning of each block also reminded participants that their task would be to respond as fast and as accurately as possible to the centrally presented target, and to ignore the flanking stimuli to the left and the right of the target. Instructions remained on the screen until a participant pressed the space key. Once a block was initiated, each trial started with the appearance of the white frame. After 500 ms, flanker-target-flanker triplets were presented at the center of this frame. The stimulus remained on the screen until the subject had responded to the target. Response times were recorded from the onset of stimulus presentation. When an error was committed, a red X appeared at the center of the white frame for 500 ms. The interval between the response or the offset of the error feedback and the onset of the white frame indicating the start of the next trial was set to 200 ms.

The identity of the flanker stimuli always differed from the identity of the target. However, on congruent trials, flanker and target stimuli were assigned to the same response, whereas the target was assigned to a different response than the flankers on incongruent trials (see “Appendix” for an overview of trial types realized in this experiment). All triplets appeared equally often.

Blocks were constructed such that in a first miniblock of four trials two of the four instructed stimuli appeared as targets (one requiring a left response, the other requiring a right response), while the other two only appeared as flankers, resulting in two compatible and two incompatible trials. Similar to Cohen-Kdoshay and Meiran (2009), this setup allowed us to test whether flankers that have never been responded to lead to a FCE in the very first trials of a new task already, and whether this early instruction-based FCE differs in size from the instruction-based FCE later on during task practice.

On half of the blocks—the instructed flanker blocks—the remaining 32 trials succeeding the first miniblock followed the same logic as the first miniblock. That is, the same two stimuli served as targets only, and never appeared as flankers, whereas the other two only appeared as flankers, but not as targets (i.e., each target and flanker appeared in 16 trials, eight times in congruent and incongruent combinations each). Which stimuli served as targets or distractors was randomly assigned to participants. On the other half of the blocks, the applied flanker blocks, all stimuli could be targets or flankers on the remaining 32 trials following the first miniblock. Thus, over the course of applied flanker blocks, participants repeatedly applied each instructed mapping (eight times each, four times flanked by congruent and incongruent distractors, respectively).

Instructed and applied flanker blocks could appear in random order, with the following constraints: the first or the second block was an applied flanker block, and the number of (the remaining) identical block types (instructed, applied) in a row could not be larger than four. These constraints were applied to discourage participants from learning (over a run of blocks) that they could effectively ignore some of the instructions on instructed flanker blocks. Note, however, that the stimuli and S-R mappings were different in each block. To maintain some control over the trial transitions, we created ten pseudorandom sequences (five for each block type) in advance that were randomly assigned to two blocks of a given flanker condition. These pseudorandom sequences allowed for complete repetitions of triplets. They ensured that all triplets would appear equally often, and that congruent and incongruent trials would follow congruent and incongruent trials with equal frequency (see “Appendix” for an overview of resulting transitions in the two types of blocks).

Participants could familiarize themselves with the task while working through a practice block of 24 trials. The practice block essentially resembled applied flanker blocks and required participants to respond to the color of colored circles. The stimulus set for the practice block (circles of varying colors) of the practice block were not re-used in the experimental blocks, and the practice block was not included in the analyses.

## Results

The first trial of each block was excluded from the analyses, as were trials with errors on the preceding trial (5.2 %). Only correct trials entered the reaction time (RT) analyses. For each participant, block type and congruency condition, RTs more than 2.5 SD above or below the mean were removed (2.3 %).

### Flanker practice

To assess the overall impact of flanker practice we tested whether an instruction-induced FCE indicating automatic activation of instructed S-R mappings can be observed with this task, and whether flanker practice leads to an overall larger FCE in the applied than the merely instructed flanker condition. Although first miniblocks could be considered part of the merely instructed condition because flankers did not appear as targets during the first four trials of each block, and hence were not practiced, first miniblocks were defined as a separate block type. This allowed us to (a) remove data from the first miniblock to compare instructed and applied flanker blocks at comparable levels of task practice,^2^ (b) analyze data from the first miniblock separately to test whether an instruction-based FCE can be observed on the very first trials of working on a newly instructed flanker task (cf. Cohen-Kdoshay & Meiran, 2009), and (c) compare first miniblocks with subsequent instructed blocks to determine whether such an early instruction-based FCE would differ from the instruction-based effect in the remainder of instructed blocks that differed in terms of task practice but not flanker practice (see next section, below). Table 2 shows the mean RTs for correct responses and the mean percentage of errors as a function of flanker condition (instructed, applied) and congruency (congruent, incongruent), separately for first miniblocks and the rest of the runs/blocks that contained either instructed or applied flankers.

**Table 2:** Mean reaction times (RT), mean % errors, and flanker congruency effects (FCE) for first miniblocks and the remainder of the blocks that contained either instructed or applied flankers. For the latter two, the table shows the overall group means as well as the means for the first and the second halves of blocks

	Instructed		Applied	
	RT	% Errors	RT	% Errors
First miniblock				
Congruent	487	5.5		
Incongruent	514	4.9		
FCE	27	−0.6		
First half of block				
Congruent	443	3.3	485	4.2
Incongruent	461	4.7	504	9.0
FCE	18	1.4	19	4.8
Second half of block				
Congruent	431	3.9	485	4.7
Incongruent	435	4.3	508	9.3
FCE	4	0.4	23	4.6
Overall (1st and 2nd half)				
Congruent	437	3.6	485	4.4
Incongruent	448	4.5	506	9.1
FCE	11	0.9	21	4.7

Merely instructed and already applied flanker conditions were compared by conducting 2 (block type: instructed, applied) × 2 (congruency: congruent, incongruent) within subjects ANOVAs, excluding trials from first miniblocks. The ANOVA of RTs yielded a significant main effect of block type *F*(1,24) = 172.5, *p* < 0.01, MSE = 408.7, indicating overall slower responses in applied than in merely instructed blocks, and a significant main effect of congruency, *F*(1,24) = 38.1, *p* < 0.01, MSE = 169.8, reflecting an overall FCE in the usual direction. Crucially, the interaction between block type and congruency was also significant, *F*(1,24) = 7.51, *p* < 0.05, MSE = 84.1. Although the FCE was significant for both flanker conditions when tested alone (*t*(24) = 4.99, *p* < 0.01 and *t*(24) = 5.38, *p* < 0.01 for instructed and applied flanker blocks, respectively), the interaction between block type and congruency suggests that the 21 ms FCE in applied blocks was reliably larger than the 11 ms FCE in instructed blocks. The results from a corresponding ANOVA on percent errors mirror the RT results. The error ANOVA also revealed significant main effects of block type, *F*(1,24) = 52.9, *p* < 0.01, MSE = 3.5, and congruency, *F*(1,24) = 49.2, *p* < 0.01, MSE = 3.9, as well as a significant interaction between block type and congruency, *F*(1,24) = 18.2, *p* < 0.01, MSE = 5.1.

Separate t tests of the FCE in first miniblocks furthermore showed that an instruction-based FCE was already present in the first four trials following a new set of instructions. This early instruction-based FCE was restricted to RTs, *t*(24) = 5.28, *p* < 0.01; it was not significant for percent errors, *t*(24) = 0.6, *p* > 0.5.^3^

### Comparing effects of task practice for applied and instructed flankers

Does task practice differentially influence the instruction-based FCE and execution-based FCE? To address this question, we first compared the FCE in first miniblocks with the FCE in (the remaining) instructed blocks that differed in terms of task practice, but not flanker practice. The 2 (block type: first miniblock, instructed flanker blocks) × 2 (congruency: congruent, incongruent flankers) within subjects ANOVA of RTs yielded a significant main effect of block type, *F*(1,24) = 347.6, *p* < 0.01, MSE = 243.1, indicating slower responses on first miniblocks compared to the remainder of instructed blocks. In line with the analyses reported in the previous section, the overall instruction-based FCE was also significant, *F*(1,24) = 38.2, *p* < 0.01, MSE = 246. Importantly, the FCE was larger on first miniblocks than on the remainder of instructed blocks, as indicated by a significant interaction between block type and congruency, *F*(1,24) = 10.8, *p* < 0.01, MSE = 161.1. The corresponding 2 × 2 ANOVA on percent errors revealed a tendency for participants to make more errors on first miniblocks than on the remainder of instructed blocks, *F*(1,24) = 3.5, p < .08, MSE = 10.1. The main effect of congruency and the interaction between block type and congruency were not significant for errors (both *p*’s > 0.2).

To investigate whether and how the observed difference between the FCEs in the applied and the instructed flanker conditions develops with task practice, we additionally carried out two types of analysis comparing instructed and applied flanker blocks. First miniblocks were excluded from both (see footnote 2). The first type of analysis included block half as a factor. In these analyses, the number of trials/responses with a given set of instructions was the same for instructed and applied flanker blocks. Note, however, that target set size was smaller in the instructed than in the applied flanker condition. Hence task practice as defined above is confounded with the number of stimulus repetitions (i.e., number of flanker-target-flanker triplet occurrences) in the two block types when blocks of equal lengths are compared. Therefore, we additionally ran a second type of analysis in which the frequency of stimulus occurrences in the two block types was matched. Specifically, we compared the FCE in complete applied flanker blocks with the FCE from trials in the first block half of instructed flanker blocks, conducting 2 (block type) × 2 (congruency) ANOVAs. This was possible because the first block half of instructed blocks contained the first eight occurrences of each target (four times flanked by congruent and incongruent distractors each). This type of analysis controls for stimulus repetitions, while the number of responses with a given set of instruction differs.

For the first type of analysis including block half as a factor, the 2 (block type: instructed vs. applied blocks) × 2 (block half) × 2 (congruency) within subjects ANOVA of RTs revealed that responses were faster on the second than on the first half of blocks, *F*(1,24) = 16.9, *p* < 0.01, MSE = 239.3, on blocks with instructed flankers than with applied flankers, *F*(1,24) = 171.5, *p* < 0.01, MSE = 823.9, and on congruent than on incongruent trials, *F*(1,24) = 37.9, *p* < 0.01, MSE = 340.7. Again, the FCE was more pronounced with applied than with instructed flankers, as confirmed by a significant interaction between block type and congruency, *F*(1,24) = 7.1, *p* < 0.05, MSE = 158.5. In addition, block type interacted with block half, *F*(1,24) = 50.9, *p* < 0.01, MSE = 108.1, indicating that participants became faster with increasing task practice in the instructed flanker condition, *F*(1,24) = 66.5, *p* < 0.01, MSE = 142.7, but not the applied flanker condition, *F*(1,24) < 1, MSE = 204.6. The interaction between block half and congruency failed to reach significance, *F*(1,24) = 2.1, *p* > 0.16, MSE = 163.9. Importantly, the three-way interaction between block half, block type, and congruency was significant, *F*(1,24) = 8.4, *p* < 0.01, MSE = 114.9: the FCE from instructed flankers decreased across block halves, *F*(1,24) = 18.2, *p* < 0.01, MSE = 67.3, whereas it did not change on applied flanker blocks, *F*(1,24) < 1, MSE = 211.5. Table 2 summarizes the mean RTs and mean percent errors in each condition. The corresponding 2 (block type: applied, instructed) × 2 block half × 2 (congruency) ANOVA with percentage of errors as the dependent variable yielded significant main effects of block type, *F*(1,24) = 51.9, *p* < 0.01, MSE = 2.7, and congruency, *F*(1,24) = 48.6, *p* < 0.01, MSE = 8.0. Congruency interacted with flanker condition, *F*(1,24) = 17.7, *p* < 0.01, MSE = 10.5, again indicating a larger FCE in applied than in instructed blocks. Block half did not reach significance, nor did it interact with other factors (all *F*’s < 1), although the numerical pattern of results in errors was in the same direction as the RT results (cf. Table 2).

**Fig. 1:**
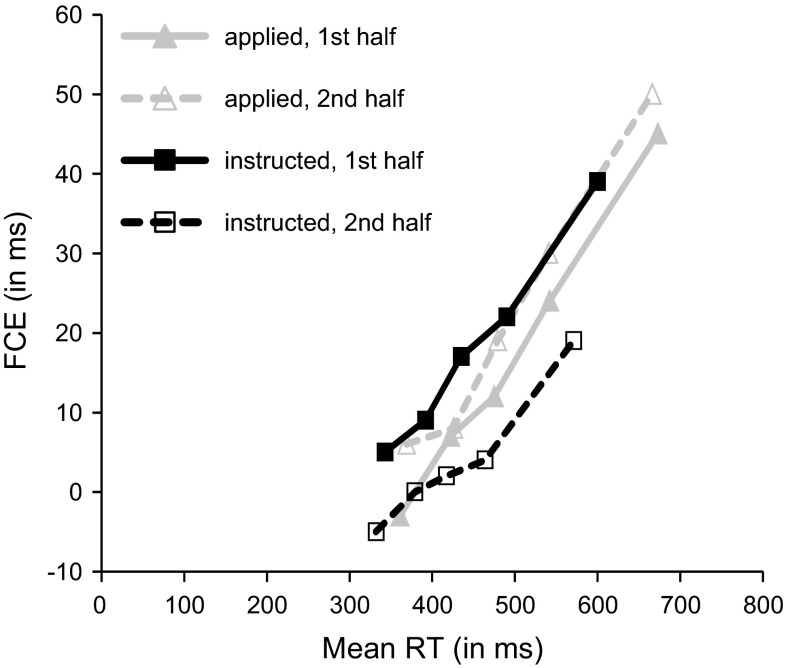
Flanker congruency effects (*FCE*) in milliseconds for the instructed and applied flanker conditions as a function of block half and mean RT (averaged across congruency conditions)

Because congruency effects such as the flanker effect often increase with overall RT (e.g., Ridderinkhof, 2002), and because instructed and applied flanker blocks in the above analysis differ regarding both, overall RT level and speed-up across block halves, we repeated the RT analysis including block half as a factor after equating mean RTs in the two block type conditions. To this end we first determined RT quintiles for each participant, block type, block half, and congruency condition. Figure 1 shows the resulting mean FCEs as a function of response speed. Inspection of Fig. 1 reveals that the size of the FCE indeed increases with overall RT. However, it becomes also evident that the instruction-based FCE is (selectively) smaller in the second block half than during the first half of blocks at comparable mean RTs. Mean RTs per cell were matched by excluding, for each participant, (a) all trials from the slowest RT quintile of both block halves in the applied flanker condition (resulting in mean RTs of 450 and 454 ms for the first and second block half, respectively), and (b) all trials from the fastest quintile in the second half of instructed blocks (resulting mean RTs were 452 ms for the first block half, and 458 ms for the second half). In the 2 (block type) × 2 (block half) × 2 (congruency) ANOVA on this subset of data, the main effect of block type was no longer significant, *F*(1,24) < 1, MSE = 1,986.2. The main effect of block half approached significance, *F*(1,24) = 3.9, *p* = 0.06, MSE = 1,194.7, reflecting the fact that mean RTs now slightly increased from 451 ms on first block halves to 456 ms on second halves. Block type and block half did not interact, *F*(1,24) < 1, MSE = 372.3. The overall FCE for applied blocks (14 ms) did no longer differ from the overall instruction-based FCE (12 ms), *F*(1,24) < 1, MSE = 673.8, but the main effect of congruency was again significant, *F*(1,24) = 37.4, *p* < 0.01, MSE = 985.8. Importantly, the three-way interaction between block type, congruency and block half, *F*(1,24) = 7.2, *p* < 0.05, MSE = 4,441 was significant, suggesting that the differential effect of task practice on the FCE in the two flanker conditions did not depend on overall RT. As in the analysis including all trials, post hoc tests showed that the size of the FCE decreased with task practice on instructed flanker blocks (from 18 ms in the first block half to 6 ms in the second half; *F*(1,24) = 11.8, *p* < 0.01, MSE = 330.9), whereas the size of the FCE in the first half (12 ms) and the second half (16 ms) of applied flanker blocks did not differ, *F*(1,24) < 1, MSE = 776.1.

In the second type of analysis the frequency of stimulus occurrences in the two block types was matched instead of the number of responses in a given block. As outlined above, this was done by comparing the FCE in complete applied flanker blocks with the FCE from trials in the first block half of instructed flanker blocks, conducting 2 (block type) × 2 (congruency) ANOVAs.

The ANOVA of RTs showed significant main effects of block type, *F*(1,24) = 103.5, *p* < 0.01, MSE = 454, and of congruency, *F*(1,24) = 41.5, *p* < 0.01, MSE = 232.2, whereas the interaction between block type and congruency was not significant, *F*(1,24) < 1, *p* < 1, MSE = 55.6. The corresponding ANOVA on the percentage of errors also yielded significant main effects of block type, *F*(1,24) = 36.7, *p* < 0.01, MSE = 5.2, and congruency, *F*(1,24) = 43.1, *p* < 0.01, MSE = 5.3. Furthermore, for errors, the interaction between block type and congruency was highly significant, *F*(1,24) = 14.3, *p* < 0.01, MSE = 5, indicating that the FCE was more pronounced for applied (4.7 %) than for instructed (1.4 %) flanker blocks.^4^ Results from a MANOVA that jointly considered RTs and errors as dependent variables mirror the results of the error analysis and revealed significant main effects of block type, *F*(2,23) = 57.5, *p* < 0.01, and congruency, *F*(2,23) = 42.9, *p* < 0.01, as well as a significant interaction between block type and congruency, *F*(2,23) = 7.4, *p* < 0.01, suggesting that the overall size of the FCEs indeed differed between flanker conditions when the number of occurrences of flanker-target-flanker triplets in the two flanker conditions was matched.

### Sequential modulation of the FCE

For the sequence analyses, we excluded first miniblocks and those trials from applied flanker blocks that instantiated transition types that did not exist for the instructed flanker blocks (see “Appendix”). This was done to maximize comparability of instructed and applied flanker conditions when performing sequential analyses.^5^ Sequential modulation of the FCE (i.e., the Gratton effect) was analyzed in the way proposed by Mayr et al. (2003), including target/response transition (repetition, change), congruency on trial *n* − 1, congruency on trial *n*, as well as flanker condition (instructed, applied) as within subjects factors. This design allowed us to disentangle the contributions to the Gratton effect in the current task of conflict adaptation, on the one hand, and of binding and retrieval of stimulus–stimulus and stimulus–response episodes (for an overview, see Verguts & Notebaert, 2009; see “Discussion” for details), on the other hand. Importantly, and as outlined in the introduction, only the conflict adaptation account predicts a larger Gratton effect for the applied than the instructed flanker condition, which should occur in both, target/response repetition and change trials.

**Fig. 2:**
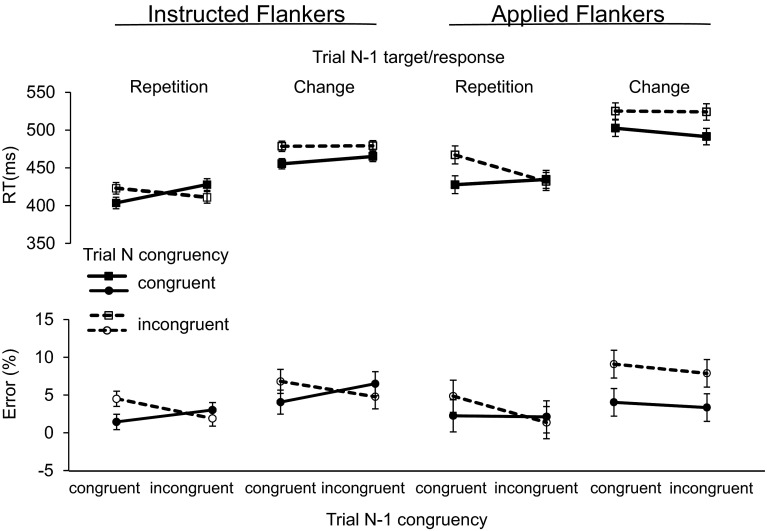
Mean RTs (*upper panel*) and % errors (*lower panel*) for instructed and applied flanker blocks as a function target/response transition (repetition, change), congruency on trial *n* − 1, and congruency on trial *n*. *Error bars* represent confidence intervals calculated according to Loftus and Masson (1994)

Figure 2 depicts the group means of RTs and errors, indicating that the Gratton effects were restricted to target/response repetition trials, and were of similar size for instructed and applied flankers. This impression was confirmed by the ANOVAs that revealed a significant modulation of the Gratton effect by target transition, while the Gratton effect interactions with flanker condition did not reach significance (see Table 3 for a complete summary of ANOVA results).

**Table 3:** Sequential modulation of the FCE

Effect	RT		% Errors	
	*F*(1,24)	*p*	*F*(1,24)	*p*
Flanker condition	81.2	<0.01	<1	>0.62
Transition	119.0	<0.01	25.4	<0.01
Congruency *n* − 1	1.7	>0.20	3.6	=0.07
Congruency *n*	41.7	<0.01	18.5	<0.01
Flanker cond. × transition	4.3	<0.05	<1	>0.46
Flanker cond. × congruency *n* − 1	14.2	<0.01	1.7	>0.20
Flanker cond. × congruency *n*	15.8	<0.01	6.3	<0.05
Transition × congruency *n* – 1	<1	>0.35	<1	>0.32
Transition × congruency *n*	14.2	<0.01	4.1	>0.05
Congruency n − 1 × congruency n (Gratton effect)	**12.8**	**<0.01**	**11.3**	**<0.01**
Flanker cond. × transition × congruency *n* − 1	<1	>0.37	< 1	>0.86
Flanker cond. × transition × congruency *n*	<1	>0.35	5.7	<0.05
Flanker cond. × congruency *n* − 1 × congruency *n*	**<1**	**>0.44**	**2.9**	**>0.09**
Transition × congruency *n* − 1 × congruency *n*	**23.0**	**<0.01**	**<1**	**>0.44**
Flanker cond. × transition × congruency *n* − 1 × congruency *n*	**1.9**	**>0.18**	**1.02**	**>0.32**

The table shows the results of the 2 (flanker condition: applied, instructed) × 2 (target/response transition: repetition, alternation) × 2 (congruency on trial *n* − 1) × 2 (congruency on trial *n*) repeated measures ANOVAs for RTs and % errors

Relevant interactions involving the Gratton effect are in bold

## Discussion

Using a flanker task with new S-R mapping instructions on each block of trials, we investigated the effects of task and flanker practice on the overall FCE, and we compared the Gratton effect with practiced as opposed to merely instructed flankers. Our results revealed an instruction-based FCE in the very first miniblock, replicating and extending findings by Cohen-Kdoshay and Meiran (2007, 2009). Cohen-Kdoshay and Meiran (2007) argued that such instruction-based FCEs are not due to inadvertently applying flanker-response mappings on some trials because they could replicate their basic findings when participants were discouraged to include flanker-based evidence when selecting a response, by presenting only incongruent trials on a practice block or by spacing targets and flankers further apart. Moreover, the instruction-based FCE in our experiment cannot be due to enhanced stimulus identification on congruent trials because we did not include congruent trials with perceptually identical targets and flankers (e.g., AAA trials). Therefore, the most likely explanation for the instruction-based FCE in our experiment holds that there was an automatic activation of the responses assigned to flankers by instruction. Automatic response activation also is the dominant explanation of FCE observed with applied mapping, suggesting functional similarities between merely instructed and already applied mappings.

### Effects of task and flanker practice on the FCE

However, our results also revealed dissociations between the two types of mapping. First, the execution-based FCE tended to be larger overall than the instruction-based FCE with comparable amounts of task practice as defined by the number of trials following a specific set of instructions. Second, the instruction-based FCE decreased with task practice: The FCE in first miniblocks was larger than in the remainder of instructed blocks, extending the findings by Cohen-Kdoshay and Meiran (2009) obtained with category-response instructions. Furthermore, and importantly, the instruction-based FCE decreased across block halves, whereas the execution-based FCE remained constant.

It could be argued that these dissociations may be due to differences in target set size between conditions. First, a larger target set is typically associated with slower responses, and congruency effects like the FCE often increase with overall RT level (e.g., Ridderinkhof, 2002). Thus, the larger overall FCEs in the applied than in the instructed flanker condition that was constant across block halves (as opposed to the instructed flanker condition where the FCE decreased) could be due to the fact that responding in the applied flanker condition was generally slower than in the instructed flanker condition, and, unlike the instructed flanker condition, did not substantially speed-up across block halves. However, although our distribution analysis revealed that the size of the FCE(s) in the present experiment indeed depended on overall response speed, the main results of the task practice analysis were replicated after equating flanker conditions in terms of overall RT: the instruction-based FCE still decreased across block halves whereas the execution-based FCE did not. Moreover, the pattern of results in errors generally mirrored the (overall) RT results.^6^

Second, differences in target set size lead to an unequal number of stimulus occurrences (i.e., triplet and hence also target appearances) when task practice is defined as the number of trials/responses under a given instruction regime. It is conceivable that, with a limited number of possible triplets, a whole triplet becomes associated with a particular response over the course of task practice. If this were the case, then the differential decline of the FCEs in the two flanker conditions across block halves could be due to the larger number of occurrences of specific triplets, and hence stronger associations between stimulus ensembles and responses, on instructed as compared to applied blocks. To rule out this possibility, we additionally compared the instruction-based FCE from trials pertaining to the first block half with the execution-based FCE including all trials in a block. In this analysis, the number of occurrences of specific stimulus (triplet) exemplars was matched. These analyses still revealed a larger FCE in the applied compared to the instructed flanker condition for errors and in the MANOVA that simultaneously considered RT and percentage errors as dependent variables. This result is in line with findings by Wendt and Luna-Rodriguez (2009) who demonstrated that learning the mappings from stimulus (target-flanker) ensembles to responses contributes little to the proportion congruent effect on the size of the FCE. The latter effect manifests itself in smaller FCEs with a high proportion of incongruent trials, and an enhanced FCE with more congruent than incongruent trials.

Taken together we conclude that neither different overall response speed nor differences in the number of times specific target-flanker ensembles appeared were the primary reasons for the observed dissociations between execution-and instruction-based flanker conditions in our experiment. Instead, we propose that the primary reason for the observed differences may lie in the functional properties of the S-R links underlying the FCE in the instructed and the applied flanker conditions. In particular, the effects of task and flanker practice observed in the current experiment were likely due to differential strengthening of S-R links. In instructed blocks, only half of the mappings were practiced and strengthened (i.e., the target-response mappings), whereas the flanker-response mappings were never applied and hence not strengthened. As a consequence response conflict resulting from activation of merely instructed S-R mappings becomes weaker with task practice, either because they are less likely to compete with the stronger activation resulting from the (target) associations based on practice (and instructions), or because merely instructed mappings that never appear as targets become weaker themselves. The latter would be the case if the associations based on instructions alone decrease in strength or if these mappings were removed from the task-set (cf. Meiran et al., 2012). In contrast, in applied blocks all S-R links become equally strengthened over the course of a block, leading to an overall larger FCE than in instructed blocks that furthermore does not decline with task practice. Viewed in the light of these findings, part of the failure to observe evidence for instruction-based automatic response activation in the Waszak et al. (2008) study might stem from selective strengthening of practiced but not instructed S-R mappings.

The differential strengthening account seems consistent with findings by recent fMRI experiments that either investigated brain activation during instruction encoding (e.g., Dumontheil, Thompson, & Duncan, 2011; Hartstra, Kühn, Verguts, & Brass, 2011; Hartstra et al., 2012) or during the first few applications of newly instructed rules (e.g., Ruge & Wolfensteller, 2010; Woolgar, Hampshire, Thompson, & Duncan, 2011). Although these studies did not actually test whether instructions were encoded in a way that allows *automatic* S-R activation from trial one they nevertheless tend to show activation of (parts of) the fronto-parietal network (e.g., Duncan, 2010) to be associated with forming and uploading task-sets in(to) (capacity-limited) working memory. Activation in the fronto-parietal network appears to decline during early task practice (Ruge & Wolfensteller, 2010; Woolgar et al., 2011). In contrast, activation seems to increase in motor-related (e.g., lateral premotor cortex) and habit-related (e.g., caudate nucleus) regions during early task practice (Ruge & Wolfensteller, 2010). Based on such findings, Ramamoorthy and Verguts (2012) recently proposed a dual process model in which a fast-learning slow-acting frontal learning system implements instructions and controls responding in the initial phase of working on a new task. The frontal system initially “guides” a slow-learning fast-acting habit-learning system that establishes direct sensorimotor links (thought to involve the basal ganglia reward learning habit system). The dual route model by Ramamoorthy and Verguts (2012) assumes that task practice shifts control from temporary frontal S-R links to qualitatively different direct sensory motor links.

Future studies need to investigate just how many executions of newly instructed mappings are needed to counteract automatic activation of already practiced S-R episodes under which conditions, whether and how applied mappings with only a few executions differ from highly over-learned mappings (also see Wolfensteller & Ruge, 2012), and whether there is an asymptote to associative strengthening. Such studies might also want to establish that initial task instructions do not overtax working memory constraints (cf., Duncan, Schramm, Thompson, & Dumontheil, 2012), for instance by testing whether instruction-based automatic effects can be observed at least early on during task practice.

### Sequential trial-by-trial modulation of instruction-based and execution-based FCEs

To better understand the functional similarities and differences between merely instructed and applied mappings, we additionally compared the sequential trial-to-trial modulations of the FCEs pertaining to the two types of mappings. These analyses revealed a Gratton effect for merely instructed mappings that did not significantly differ in size from the effect for applied mappings. Furthermore, the Gratton effect for both types of mapping was (similarly) restricted to target/response repetition trials. These results do not support the conflict adaptation account according to which cognitive control adjustments should depend on the amount of conflict on the previous trial (Davelaar & Stevens, 2009; Wendt et al., 2013)—irrespective of whether targets/responses repeat or alternate. Provided that response conflict was more pronounced in the applied than in the instructed flanker condition (as argued above), one should therefore have observed a more pronounced Gratton effect in the former than in the latter condition, independent of target/response transition. Instead, the pattern of results is consistent with the repetition priming account of the Gratton effect (Mayr et al., 2003). The repetition priming account holds that the Gratton effect is mainly due to differential priming of stimulus (i.e., target and flankers)-response episodes in target/response repetition trials. In particular, complete repetitions of S-R episodes that only occur for congruent trials following congruent trials and incongruent trials following incongruent trials are assumed to be processed faster than partial repetitions that occur in compatible trials following incompatible trials and vice versa (see “Appendix”). Thus, according to the repetition priming account the Gratton effect should only show up for target repetition trials, and should not depend on the amount of response conflict on the previous trial.

At first sight, this result may seem surprising, given that Mayr and Awh (2009) demonstrated item-general conflict adaptation effects occurred in the first blocks of their experiment, but not after extended practice. They suggested that conscious deliberate regulation attempts lead to conflict adaptation effects when working on new tasks, but not later on when instructed mappings have been “sufficiently” practiced. Therefore, one might have expected evidence for item-general top-down conflict adaptation at least in applied flanker blocks of our experiment that involved only few executions of newly instructed mappings.

One reason for not observing item-general conflict adaptation in our study could be that our experimental settings were unfavorable. First, some findings (Verbruggen, Notebaert, Liefooghe, & Vandierendonck, 2006; Notebaert & Verguts, 2006) indicate that conflict adaptation effects might depend on congruent trials with and without flankers that are identical to the target. Our experiment, unlike Mayr et al. (2003) or Verbruggen et al. (2006), did not include trials with identical congruent flankers. Second, conflict adaptation effects appear to be sensitive to the timing of events (Egner, Ely, & Grinband, 2010; Ullsperger, Bylsma, & Botvinick, 2005). The timing of events in our experiment might not have been favorable. Clearly, more work is needed to better understand the role of response conflict in influencing sequential modulations of instruction-based and early execution-based flanker effects. Future studies should include manipulations that allow a better distinction between different accounts of the Gratton effect (e.g., item specific congruency effects, proportion congruent trials) and might want to give the conflict adaptation account a fairer chance by including identical flanker trials and by adapting the timing of events within trials.

## Conclusion

We found a merely instructed flanker effect with instructed S-R mappings that was already present in the very first trials. However, the FCE from merely instructed flankers decreased with task practice whereas the execution-based FCE did not, leading to an overall larger execution-based FCE. This dissociation can best be explained by associative strengthening when repeatedly applying instructed S-R mappings. Associative strengthening leads to more response conflict from flankers in the applied than the merely instructed flanker condition.

## Appendix

Examples of trials and transitions for a mapping that assigns the letters A and D to left key responses, and the B and C to right key responses, where only the letters D and B can appear as targets in the instructed flanker condition (only trials requiring left-hand responses on trial *n* depicted). The first letter triplet in each pair of triplets depicts distractor-target-distractor combinations that can occur on trial *n* − 1; the second triplets represent example stimuli on trial *n*. Bold font indicates realizations of trials/transitions that can appear in both, the instructed and the applied flanker condition, normal font indicates realizations of transition types that exclusively occurred in the applied condition.

**Table:**

Transition type	Target repetition				Target alternation			
	Flanker repetition		Flanker alternation		Flanker repetition		Flanker alternation	
	Response repetition	Response alternation	Response repetition	Response alternation	Response repetition	Response alternation	Response repetition	Response alternation
Congruet–congruent	**ADA–ADA** DAD–DAD	–	–	–	–	–	ADA-DAD^a,b^ DAD-ADA^a,b^	**CBC-ADA** CBC-DAD BCB-ADA BCB-DAD
Congruent–incongruent	–	–	**ADA-CDC** DAD-BAB	–	–	**CBC-CDC** BCB-BAB	ADA-BAB^a,b^ DAD-CDC^a,b^	CBC-BAB^a^ BCB-CDC^a^
Incongruent–congruent	–	–	**CDC-ADA** BAB-DAD	–	–	**ABA-ADA** DCD-DAD	CDC-DAD^a^ BAB-ADA^a^	ABA-DAD^a,b^ DCD-ADA^a,b^
Incongruent–incongruent	**CDC–CDC** BAB–BAB	–	–	–	–	–	CDC-BAB ^a^ BAB-CDC^a^	**ABA-CDC** DCD-BAB ABA-BAB^b^ DCD-CDC^b^

^a^Transition types that were excluded from the sequence analyses because they only occurred in applied flanker blocks

^b^Negative priming trials

## Footnotes

1 We acknowledge the possibility that the effects of practice are not mediated by the strengthening of the associations between (conceptual) stimulus and response codes set up by instructions, but instead by the formation of other types of representations (e.g., by forming and strengthening direct links between sensorimotor codes that bypass the instructed links; (Ramamoorthy & Verguts, 2012). Nevertheless, we will use “strengthening of S-R associations” as shorthand for all possible changes in representations that might mediate the effects of practice.

2 Removing trials from the first miniblock when comparing instructed and applied flanker blocks was necessary in order to avoid a confound between flanker and task practice. This is because first miniblocks without flanker practice preceded both types of block (see “Methods” for details).

3 Including first trials of each block/miniblock led to substantial increases of mean RTs in first miniblocks, without affecting our conclusions based on results that excluded first trials. Specifically, the mean scores for first miniblocks including first trials were 509 ms (4 % errors) and 541 ms (4 %) in the congruent and incongruent conditions, respectively, when first trials were included.

4 When only those triplets in applied blocks were included in the analysis that had also occurred in the first miniblock, mean RTs/percent errors were 488 ms/4.3 % and 502 ms/9.4 % in the congruent and incongruent applied flanker conditions, respectively, leading to the same significances as the ANOVAs including all triplets.

5 Including all existing trial transitions of the applied flanker condition when analyzing the Gratton effect does not lead to different conclusions than the analyses based on the reduced set of transitions. Likewise, further excluding negative priming trials in which flankers on trial *n* − 1 became targets on trial *n* from the reduced applied flanker set (see “Appendix”) did not notably change the pattern of results reported here, either.

6 In a similar vein, Cohen-Kdoshay and Meiran (2009) argued that the larger FCE in first miniblocks than the remainder of their instructed flanker blocks did not solely depend on speed-up with task practice. In a separate study they demonstrated that a secondary task substantially increased RTs in their instructed flanker task while at the same time eliminating the FCE.
